# Effect of organizational citizenship behavior on family-centered care: Mediating role of multiple commitment

**DOI:** 10.1371/journal.pone.0204747

**Published:** 2018-09-26

**Authors:** Mustafa Mahooti, Parvaneh Vasli, Esmail Asadi

**Affiliations:** 1 Department of Management and Economics, Science and Research Branch, Islamic Azad University, Tehran, Iran; 2 School of Nursing and Midwifery, Shahid Beheshti University of Medical Sciences, Tehran, Iran; Nord University, NORWAY

## Abstract

Family-centered care is one the most important indicators of high-quality care. The organizational citizenship behavior and commitment can enhance the quality of healthcare. This study aimed to investigate the effect of the organizational citizenship behavior on family-centered care considering the mediating role of multiple commitment. This descriptive study was conducted on 237 nurses working in pediatric and infant units of hospitals in Tehran city, Iran. The subjects were selected using the convenience sampling method. Data collection was performed using the Organizational Citizenship Behavior Scale, Perceived Family-Centered Care Staff Questionnaire, Affective Commitment Scale, and Commitment to the Supervisor Scale. The SPSS v.22 and SEM-PLS v.2 software were used for data analysis. Results were extracted in the form of a standard model and fitted for indices pertaining to the measurement and structural models. Accordingly, the organizational citizenship behavior had a direct effect on family-centered care (β = 0.19, t = 2.39). Moreover, multiple commitment including commitment to the leader and commitment to the organization had indirect weak and moderate effects on the relationship between the organizational citizenship behavior and family-centered care, respectively. An inverse association was reported between commitment to the leader and family-centered care. Furthermore, the organizational citizenship behavior predicted family-centered care by 70% considering the mediating role of multiple commitment. Therefore, family-centered care as an indicator of high-quality care can be improved through enhancing the organizational citizenship behavior and organizational commitment among Iranian nurses working in pediatric wards.

## Introduction

Similar to other service-oriented organizations, healthcare organizations face with challenges in the process of providing high-quality services [1]. The Institute of Medicine (IOM) has highlighted the importance of high-quality care in the past decade [2]. In many healthcare organizations, nurses account for 40–60% of healthcare staff [3]. Since nurses mostly contact patients, the quality of nursing care influences the general perception of people about the hospital and indirectly influences its performance [4, 5]. The IOM has offered extensive discussions regarding the definitions and methods of provision of nursing care [6]. This institute introduced patient and family-centered care (PFCC) as one of the six important elements of high-quality care, based on the effective relationship between healthcare specialists and patients and their families [2]. Also, the quality of hospital services not only depends on technical care services, but also is influences by interpersonal relationships [6]. In fact, the concept of family-centered care (FCC) presents a customer-centered model in the healthcare context considering the specific needs of patients and their families, and the need for treating them with respect and dignity [7]. Nowadays, the enhancement of customer-centered behaviors by nurses and the improvement of their relationships with patients and their families are emphasized to improve the quality of services. On the other hand, the healthcare recipients evaluate the quality of healthcare based on personnel behaviors [8].

In addition to FCC as an important concept in the provision of healthcare, organizational citizenship behavior (OCB) is a facilitator for the enhancement of healthcare in the healthcare system. The improvement of OCB can lead to the domination of a positive organizational atmosphere for enhancing the quality and quantity of healthcare services, satisfaction of patient and staff, collaboration, teamwork, and the efficiency and effectiveness of the healthcare system [9]. Nursing needs to be equipped with ethical and practical guidelines to make them responsible for maintaining the personal behavior standards and conform to behaviors known as OCB [10].

In addition to above two constructs, commitment influences the quality of healthcare [11] and behaviors at the organizational level. Committed staff feel more satisfied with their job and endeavor to achieve organizational objectives [12]. Han and Chung believe that nurses’ commitment is an essential prerequisite to reduce conflict, fatigue, job turnover, and can enhance commitment to patients’ health [13].

This study was conducted to investigate the effect of OCB on FCC considering the mediating role of multiple commitment. While the relationships between commitment, OCB, and customer orientation (recognized as FCC in the healthcare context) have been studied, no study is available on the effect of OCB on FCC considering the mediating role of commitment. Therefore, this study was conducted to fill this knowledge gap. The results of this study can be used to improve the quality of care delivered to patients in healthcare services.

## Theoretical background

### Family-centered care

FCC is a concept related to customer orientation [14]. Customer orientation is defined as a shift in organizational values, attitudes, presumptions, and commitment toward a mutual customer-organization relationship. It is a set of beliefs in favor of customer interests and needs. It also refers to identification, analysis, understanding, and response to customer needs [15]. According to Saxe and Weitz, customer-oriented behaviors (COBs) refer to helps to a customer for making a decision about services [16]. They are required for meeting the customer needs and making him/her satisfied. In the healthcare context, more precise and high-quality care is needed to meet patients’ medical needs [17]. COBs are defined as the personnel tendency to satisfy the patients through providing patient-friendly services [18]. In nursing, COBs are a degree of nurses’ understandings of patients’ needs and demands, which enable them to find a solution to patients’ problems or help them deal with their problems [17]. The provision of patients with high-quality care needs strengthening the individual/family-oriented attitude, as well as effective customer-oriented care skills [19].

FCC is based on customer orientation [20] and refers to the adoption of an innovative strategy in pediatric healthcare planning, delivery, and assessment, and can lead to the mutual cooperation of parents, families, and healthcare workers (HCWs) with an emphasis on the needs of parents and families [21]. FCC as a part of PFCC provides parents with information and knowledge about the current condition of their children and related care programs. It also supports their role, and involves them in clinical decision-making to build up their trust in the team of healthcare specialists [22–24]. FCC as a healthcare standard is associated with positive outcomes such as efficiency enhancement, timely discharge, and greater satisfaction of parents and staff [25].

#### Organizational citizenship behavior

OCB was introduced by Bateman and Organ in 1983 and was refined by other researchers [26]. Organ [27] defined OCB as “individual behavior that is discretionary, not directly or explicitly recognized by the formal reward system, and in the aggregate promotes the efficient and effective functioning of the organization” (p. 3).

This behavior can be considered a facilitator of high-quality healthcare [9] in the following general forms: ‘interpersonal OCB (IOCB)’ and organizational OCB (OCBO). The first one refers to relationships between colleagues and can improve one’s individual and organizational performance. Organizational OCB (OCBO) refers to behaviors toward the organization and organizational activities and can affect the general performance of the organization [28]. The most classical dimensions of this concept are civic virtue, altruism, conscientiousness, sportsmanship, and courtesy, which can carry different meanings to HCWs [29].

Altruism helps HCWs to achieve organizational objectives. Conscientiousness makes HCWs avoid unnecessary discussions or long individual telephone calls, and ensures their timely presence at the patient’s bed and handle required tasks. Sportsmanship prevents HCWs from looking for problems inside or outside of the caring department and persuade them to spend more time for achieving the organizational productivity. Doing what is the best for patients and co-workers improves courtesy. Civic virtue refers to participation and concern with respect to the organizational life [30].

#### Multiple commitment

Commitment is defined as a “force that binds an individual to a course of action of relevance to one or more targets” [31]. Committed staff are more likely to engage in desirable behaviors such as high performance and motivation, and create value for the organization [32]. Reichers in a review study on the nature of organization, research, and role theory showed that multiple commitment should be considered more accurately and meaningfully [33]. Recent studies argue that commitment should be investigated from different perspectives such as organizational and supervisory. Staff mainly engage in separate exchange relationships with the organization to which they belong and the supervisor is in charge of monitoring their performance [32, 34].

This study considered commitment in a two-dimensional multiple foci form: commitment to the organization (CTO) and commitment to the leader (CTL). CTL importance is due to the fact that leadership is one of the most important predictors of commitment [32]. Previous nursing studies have emphasized this type of commitment and have investigated the role of nurse-supervisor relationship in relation to commitment [35].

In addition to CTO, CTL as organizational commitment (OC) is one of the most important concept in organizational behaviors and human resource management [36]. Porter et al. believe that organizational commitment is the strength of an individual’s identification and involvement in an organization [37].

Organizational commitment has three dimensions as affective, continuance and normative. Affective commitment refers to an employee's emotional attachment to identification with and involvement in the organization. Continuance commitment refers to commitment based on the costs that employees associate with leaving the organization. Normative commitment refers to an employee’s feelings of obligation to remain in the organization [38]. Organizational commitment is a mental status affected by different types of employee-organization relationship [39]. People with high levels of CTO seek out organizational goals without considering their personal interests [36].

### Hypothesis

#### Social exchange theory

This study was based on the Social Exchange Theory (SET) as a conceptual paradigm in organizational behaviors [40]. The SET theorists believe that interpersonal behaviors are oriented via social exchange principles. The cornerstone of this hypothesis is the mutual nature of social relationships as a form of interaction in which two people voluntarily offer benefits to each other and accept gained benefits as reward. In other words, people step into exchange relationships expecting benefits from them. Exchange is a concept that indicates how people in social interactions influence each other through the mutual exchange of rewards [41]. The SET can be used to describe the relationship between OCB, commitment, and FCC. Accordingly, individuals with a good perception of their organization behave in ways to benefit the organization and co-workers and show their commitment [42]. On the other hand, SET argues that employees with a helpful behavior such as OCB may show other helpful behaviors such as customer orientation, which can be due to values attached to the socialization process [43].

#### Relationship between organizational citizenship behavior and commitment

When an employee has a relatively positive perception of the organization, he/she would benefit it in the most effective and economic way, and OCB is one the best methods of showing benevolence [5]. Therefore, SET supports the relationship between OCB and commitment. This theory claims that individuals with a satisfactory perception of their organization behave in ways to benefit the organization and co-workers and show their commitment [42]. Committed individuals to the organization tend to have a friendly relationship with their coworkers and perform their organizational tasks desirably [13].

Commitment and CTO in particular reflect individuals’ attempts to achieve organizational goals. CTO as a multidimensional concept has positive correlations with OCB. Individuals with CTO spend more time on their organizational tasks and are more organized in their job [44]. Investigation of the commitment of the nursing staff to their organization and OCB at the same time is very important [45].

There are various studies on the relationship between these two concepts in nursing. For example, Huang et al. found a multidimensional analysis of such variables as job satisfaction, organizational commitment, and organizational commitment behavior [5]. Duarte also examined the relationship between OCB and professional commitment [10]. Also, this relationship was investigated in the current study through putting forward the following hypotheses.

H1a: OCB positively affects CTO.
H1b: OCB positively affects CTL.

#### Relationship between commitment and family-centered care

The organization’s achievement for the improvement of performance needs a workplace setting in which nursing staff are committed [45]. The frontline staff can influence the customers’ perception, attitude, and assessment of the organization [46]. Those individuals who are strongly committed to their organization spend more time on their organizational tasks and are more organized in their job [36, 44]. Strong organizational commitment encourages staff and makes them familiar with organizational goals and customer’s satisfaction through exhibiting better customer-oriented behaviors [12]. Organizational commitment can directly influence the quality of care and indirectly affect the healthcare organization [11].

The authors of this study did not find any studies focusing on the relationship between commitment and FCC. However, some studies considered the relationship between commitment and FCC, i.e. customer orientation. Hsu et al. investigated the relationship between organizational commitment and customer-centered behavior among nurses [47]. Another study was conducted in Korea on OC, job satisfaction, tendency to turnover, and customer-centered behavior of the nurses [48]. Baird, Tung, and Yu studied the senior and middle nursing managers in 487 hospitals across Australia to investigate the relationship between staff’s OC and operational performance and patient care [12]. Parallel to the above studies, two other hypotheses on commitment (CTL and CTO) and FCC were formulated as follow:

H2a: CTL positively affects FCC.
H2b: CTO positively affects FCC.

### Relationship between family-centered care and the organizational citizenship behavior

Clinical nurses account for the majority of HCWs in healthcare organizations. They are on the frontline when it comes to dealing with patients and their perceptions and behaviors affect the value of service delivered by the healthcare system to patients [9]. OCB is an important factor concerning the behavior, attitude, and interaction of employees influencing the provision of high-quality care [15]. Previous studies assessed the effect of employees’ OCB on organizations and coworkers, and acknowledged the significant effect of OCB on customers. This form of OCB is specifically relevant to the extra-role of staff performance in the provision of services to customers. This reflects the customer-directed extra-role of performance and greater attempts of employees in the delivery of service [49].

Nursing OCB is known as an important skill to promote the welfare and quality of care delivered to patients [50]. While the specificity and sensitivity of high-quality healthcare increases the need for OCB [1], the significant effect of OCB on the quality of healthcare, with PFCC as one of its indicators has received less attention [29, 51]. The relationship between OCB and FCC can be explained through applying the SET theory. According to this theory, OCB is altruism-oriented. On the other hand, nurses develop altruistic or prosocial characteristics through understanding the concept of PFCC as these two concepts are correlated together [18].

Moreover, customer orientation can be defined as staff’s ability and tendency to help customers to participate in behaviors with the aim of meeting their needs. In fact, customer orientation can be considered a form of OCB (customer-directed OCBs) in which the real customer-centered behaviors depend on the employees’ freedom in fulfilling far beyond the customer satisfaction level [52].

**Fig 1:**
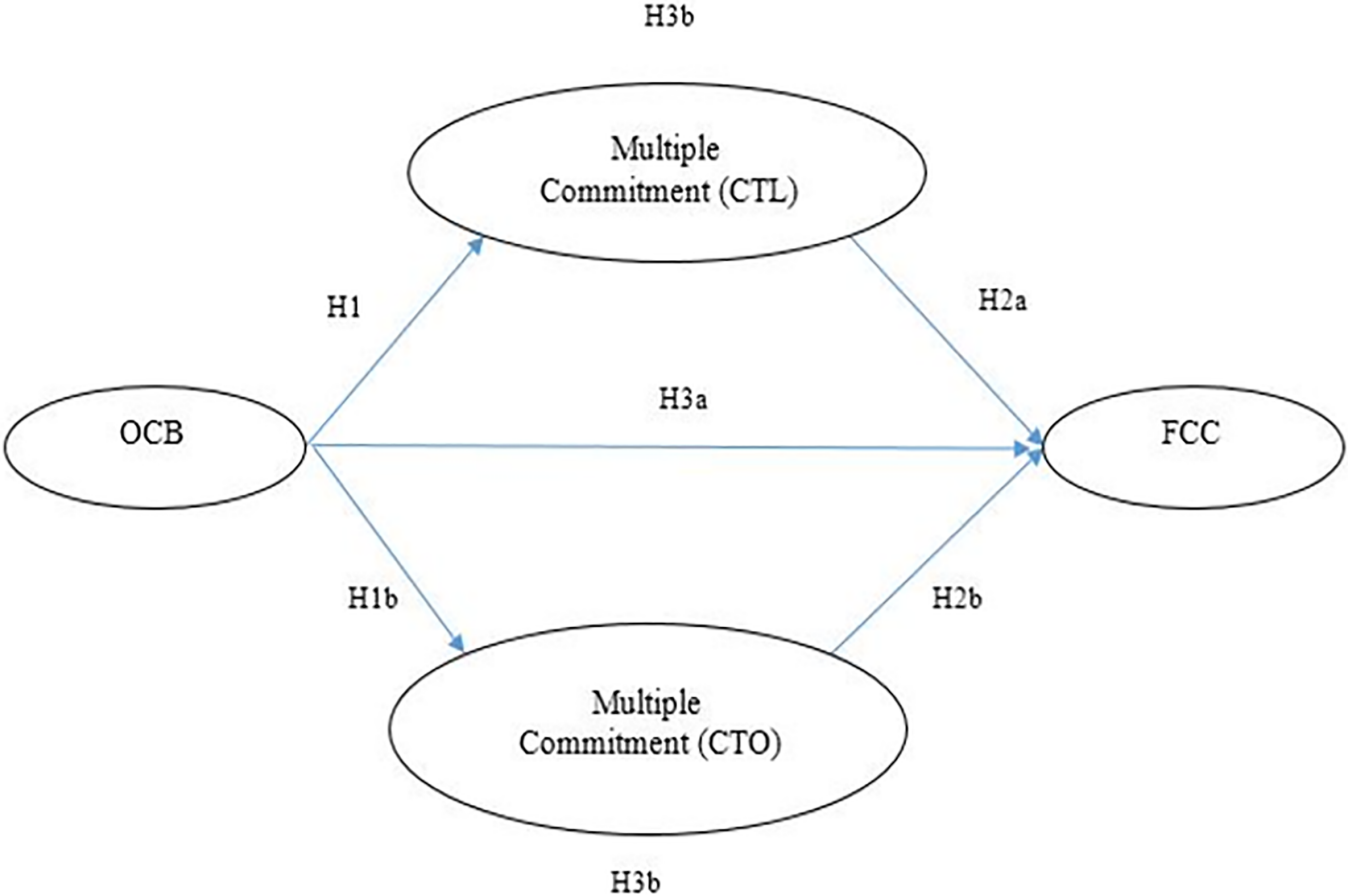
Conceptual model of the study.

Accordingly, OCB can be connected to a customer-centered behavior known as FCC in the current study. Lan, Chen, and Chang investigated the relationship between OCB, FCC, and patient satisfaction [18]. Also, Chien et al. studied the relationship between OCB and customer-centered behavior of nurses [17]. Dastyari and Shahabi assessed the relationship between OCB and customer loyalty and service quality [29]. Accordingly, the final hypotheses are as follows:

H3a: OCB positively affects FCC. Therefore, a conceptual model was developed (Fig 1). In addition to investigating the direct effect of OCB on FCC, this study eventually intended to investigate this effect indirectly considering the mediating role of multiple commitment (CTO and CTL). Therefore, the last research hypothesis is as follows:
H3b: Multiple commitment as a mediating variable has an indirect effect on the relationship of OCB with FCC.

## Material and methods

### Design and samples

This applied, descriptive, cross-sectional survey used a structural equation modeling (SEM) to investigate the effect of OCB on FCC, considering the mediating role of multiple commitment (CTL and CTO). It used a combination of regression and factor analysis methods and was executed in Smart PLS. This analysis technique is consisted of measurement and structural model fit [53].

The research was conducted in November 2016 to October 2017 on nurses working in the neonatal and pediatric wards of five educational general hospitals and a children`s hospital affiliated with Shahid Beheshti University of Medical Sciences (SBMU) in Tehran city, Iran. The sample size was estimated to be 214 based on the Morgan and Krejcie’s table. Considering samples’ drop outs and incomplete data collection, the sample size was increased to 20% (257). Finally, 237 nurses filled out instruments for data analysis. Inclusion criteria were willingness to partcipate in the study, a minimum work experience of one year in pediatric and infant units, and bachelor's degree or above in nursing. They were selected using a convenience sampling method.

### Procedures and data collection

The researchers attended pediatric wards of the hospitals in different days and various work shifts for data collection. They filled out instruments in a predefined time period.

### Measurements

#### Organizational citizenship behavior

OCB was measured using the Farsi version of the standard 21-item Podsakoff’s scale. It measured five concepts including altruism (4 items), conscientiousness (5 items), sportsmanship (4 items), and courtesy (4 items), and civic virtue: (4 items). Scoring was based on a Likert scale anchored by 1 (‘very low’) to 5 (‘very high’). Items 6, 9, 10, 11, 12, and 14 were scored inversely. The maximum and minimum scores were 21 and 105, respectively. Reliability of this scale was confirmed in other studies [54].

#### Multiple commitment

In this study, multiple commitment was measured using two scales. The 5 items of 8-item Affective Commitment Scale (ACS) was selected and used to measure CTO. It was developed by Allen and Mayer in 1990. The Farsi version of this scale has been used by Iranian researchers, and its validity and reliability has been confirmed [3].

Moreover, the 5 items of 6-item Commitment to the Supervisor (CS) scale, developed by Vandenberghe et al. was selected and used to measure CTL [34]. After obtaining permissions to use this scale, it was translated to Farsi through a forward-backward procedure and its validity and reliability was confirmed. Responses were on a 5-point Likert scale anchored by 1 (‘strongly disagree’) to 5 (‘strongly agree’).

#### Family-centered care

This was measured using the Perceived Family-Centered Care-Staff (PFCC-S) scale. It was developed by Shields and Tanner (2004) and was used in pediatric hospitals in Australia. The scoring system of this 21-item scale was based on a 4-point Likert scale anchored by 1 (‘never’) to 4 (‘always’). The PFCC-S measured three concepts as ‘respect’ (items 1 to 6), ‘participation’ (7 to 17), and ‘support’ (18 to 21). This instrument was used for the first time in Iran after translation using the forward-backward procedure and after confirming its validity and reliability [55].

#### Validity and reliability

For validity, the opinions of an expert panel consisting of 10 faculty members of nursing and midwifery and 10 nurses working in the pediatric and infant units were sought. All items were investigated in terms of quality and face validity. The content validity ratio (CVR) for each item was measured to assess items’ significance. Moreover, the content validity index (CVI) helped investigate the relevance of items [56]. The CVIs for the CTO, CTL, OCB, and FCC-P were reported as 0.79, 0.86, 0.79, and 0.82, respectively.

The Cronbach’s alpha was used to assess reliability and internal consistency of the questionnaires. The questionnaires were administered to 20 non-participating nurses and the Cronbach’s alpha coefficient was measured. The Cronbach’s alpha coefficients for the CTO, CTL, OCB, and FCC-P were reported as 0.75, 0.89, 0.85, and 0.79, respectively.

### Data analysis

To examine the research model, the Partial Least Squares-Structural Equation Modeling [PLS-SEM) was used. According to Hair et al., the PLS-SEM is a regression-based technique that explores the linear relationship between some independent variables and one or more dependent variables, It also, measures a grid of relationship between structures, structures and their measures [53].

Considering that the primary assumption for SEM data analysis was the normality of data, it was investigated using the Kolmogorov-Smirnov test (Table 1). Data analysis was conducted using the SPSS v.22 software for describing demographic variables. The mean and standard deviation of the independent variables were analyzed using the PLS v.2 software.

**Table 1:** Kolmogorov-Smirnov test for research variables.

Variable	Test statistic	p-value
OCB	0.04	0.20
CTL	0.06	0.06
CTO	0.07	0.1
FCC	0.05	0.07

### Ethical considerations

This study was approved by the Ethical Committee affiliated with SBMU (decree code: IR.SBMU.PHNM.1395.443). The study was conducted after obtaining the permission from hospital authorities. Before obtaining the written informed consent, they were informed about the research aim, anonymity and confidentiality, voluntary participation, and right to withdraw from the study.

## Findings

The mean age of the subjects was 33.7±7.1 years. The majority of them were married (66.2%) and had no children (49%). In terms of education level, 91.5% had bachelor's degree and 8.5% had master’s degree. Furthermore, 6% of them were head nurses and 94% were general nurses. Also, 61.2% were official staff and 75.5% worked in rotating shift. The majority of the staff had less than 5-years’ work experience (42.6%) with the mean work experience of 7.9±6.2 years (Table 2).

**Table 2:** Demographic characteristics of the subjects.

Demographic characteristics	n	%
***Age***		
20–24	16	6.7
25–29	56	23.6
30–34	60	25.3
35–39	52	22
40–44	28	11.3
≥45	25	10.6
***Marital status***		
Single	76	32
Married	157	66.2
Divorced	3	1.3
Widow	1	0.4
***Number of children***		
No child	116	49
One child	63	26.5
Two children	52	22
More than two children	6	2.5
***Education level***		
Bachelor degree	217	91.5
Master degree	20	8.5
*Job title*		
Head nurse	14	6
Staff nurse	223	94
***Employment status***		
Permanent recruitment	145	61.2
Contractual	19	8
Conventional	33	14
Two-year recruitment	40	16.8
***Work shift***		
Day	34	14.4
Night	24	10.1
Rotating	179	75.5
***Experience***		
≤5	101	42.6
6–10	68	28.7
11–15	35	14.8
16–20	23	9.7
≥20	10	4.2

For data analysis using the SEM-PLS, the measurement and structural model fit should be first examined to ensure their utility. According to Vinzi et al. the measurement model examined the assumed relationship between indices and latent constructs, but the structural model investigated the assumed path between exogenous latent constructs (independent) and endogenous ones (independent) [57].

### Measurement model

#### Indicator reliability

The indicator reliability specified which part of the indicator’s variance was explained by the underlying latent variable [57]. Reliability was examined using factor loadings calculated by the correlation of a construct and its indices. According to Hair et al., a factor is valid for a construct, if its loading exceeds 0.4; otherwise, the inappropriate index should be removed for model improvement [53]. In this study, the factor loading of all constructs was higher than 0.4. As a result, none of the OCB and FCC constructs were removed from the conceptual model. The general results concerning the reliability of OCB and FCC were separately presented in Table 3.

**Table 3:** Factor loadings for the measurement model.

Variables		Factor loading	Significant Coefficient	Result
OCB	Altruism	0.780	20.770	Confirmed
	Civic Virtue	0.611	11.320	Confirmed
	Conscientiousness	0.565	8.826	Confirmed
	Courtesy	0.736	18.951	Confirmed
	Sportsmanship	0.691	14.116	Confirmed
FCC	Respect	0.763	20.476	Confirmed
	Participation	0.920	63.629	Confirmed
	Trust	0.828	32.577	Confirmed

### Construct reliability

For construct reliability, the Cronbach’s alpha coefficients and composite reliability methods were used. Composite reliability of higher than 0.7 indicated the appropriate internal consistency of the measurement model. It is worth noting that the composite reliability was a better criterion than the Cronbach's alpha in modeling structural equations [57]. The composite reliability coefficients for the CTO, CTL, OCB, and FCC were reported as 0.83, 0.77, 0.80, and 0.86, respectively. According to Hair et al., the reliability value of reliability tests like composite reliability should be between 0.7 and 0.95 for all organizations [53]. Therefore, reliability of all variables was confirmed (Table 4).

**Table 4:** Indicators of measurement and the structure model.

Variable	Average Variance Extracted (AVE)	Cranach’s Alpha	Composite Reliability (CR)	R2	CV Red.	Correlation of constructs			
						(1)	(2)	(3)	(4)
OCB (1)	0.77	0.79	0.83		0.22	1			
CTL (2)	0.73	0.70	0.77	0.59	0.31	0.70	1		
CTO (3)	0.71	0.71	0.80	0.18	0.09	0.42	0.29	1	
FCC (4)	0.79	0.82	0.86	0.70	0.18	0.40	0.22	0.83	1

### Convergent validity

Convergent validity was a measure of the model fit. The average variance extracted (AVE) showed the degree of correlation between a construct and its indices, with a greater fit being achieved with a stronger correlation [53]. In this study, the AVE of all organizations was higher than 0.5 (Table 4).

### Discriminant validity

The Fornell-Larcker criterion was used for the assessment of convergent validity. This method compared the root square of AVE with the correlation of latent variables. The square root of AVE of each latent variable should be greater than correlations between latent variables [53]. In the present study, the convergent validity of the model was confirmed (Table 4).

### Structural model

This study used significant coefficients (t-value), coefficient of determination or R^2^, cross-validated redundancy measure or Q^2^, effect size or F^2^, and goodness of fit or GOF [53, 58] to fit the structural model.

### Significant coefficients (t-value)

**Fig 2:**
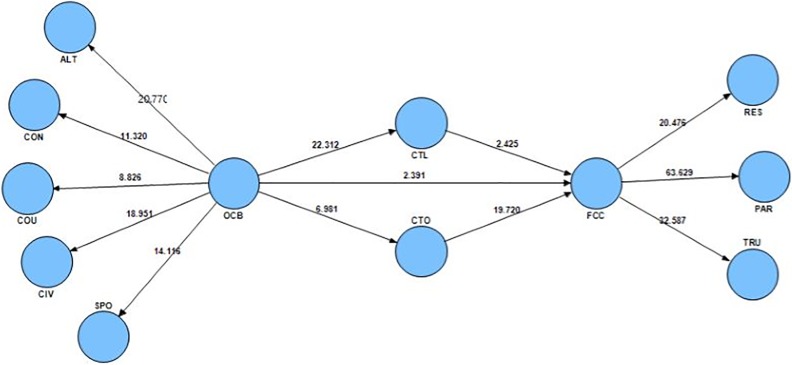
T-value for an estimate path coefficient meaningful in model.

The most basic criterion to assess the relationship between the constructs was significance coefficients (Z or t-value). If the values of these coefficients exceed 1.96, the accuracy of the relationship between constructs was confirmed at the significance level of 0.95 [53]. According to Fig 2 and Table 5, all coefficients were higher than 1.96 confirming the significance of all paths and appropriateness of the structural model.

**Table 5:** Results for the study paths.

Paths	β	t-value
OCB to CTL	0.77	22.31*
OCB to CTO	0.42	6.98*
CTL to FCC	-0.16	2.42*
CTO to FCC	0.80	19.72*
OCB to FCC	0.19	2.39*

* p≤ 0.05

### Coefficient of determination (R^2^)

**Fig 3:**
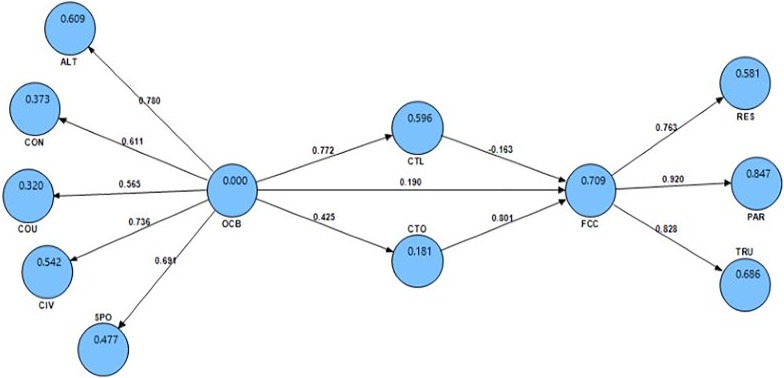
Structural research model in the standard estimation mode.

R-squared (R^2^) as the predictive power of internal constructs varied between 0 and 1: R^2^ values of 0.67, 0.33, and 0.19 could be respectively described as strong, moderate, and weak [59]. In the current study, OCB could respectively predict CTO and CTL weakly (R2 = 0.18) and moderately (R2 = 0.59). In addition, OCB provided a strong prediction (R^2^ = 0.7) of FCC considering the mediating role of CTL and CTO. These values were indicatives of relatively good model fit (Fig 3 and Table 4).

### Cross-validated redundancy measure (CV-Red)

The Q^2^ or CV-Red was used to investigate the predictive relevance of the research model. According to Henseler et al., models a with a Q^2^ value greater than zero had predictive relevance [60]. Based on Table 5, CV-Red for all constructs was larger than zero. As a result, the model had a good predictive relevance quality (Table 4).

### Effect size (F^2^)

As another indicator, F^2^ determined the strength of the relationship between the model constructs and indicated the predictive relevance of an exogenous construct for an endogenous one. This value was obtained by the following equation: F^2^ values of 0.02, 0.15, or 0.35 and can be respectively described as low, moderate, and high [53].

$$f^{2}={(R}^{2}included-R^{2}excluded)/(1-R^{2}included)$$

Using the following equation, the effects of the CTL and CTO on OCB and FCC were reported as 0.005 and 0.3 indicating the modest and moderate effects of these two variables on the mentioned relationship (Table 6).

**Table 6:** Effect size of the CTL and CTO as the mediators of the model.

Variables	Effect size	Level
CTL	0.005	Small
CTO	0.308	Medium

### Goodness of fit (GOF)

GOF is an index for the general investigation of the model and is obtained using the following equation: GOF values of 0.01, 0.25, and 0.35 could be respectively described as weak, moderate, and strong [60].

$$\sqrt{AveR2\times (Ave\;of\;communalities)}$$

The GOF for this model was obtained as 0.32. Therefore, the obtained value (0.57) showed that the model had an appropriate goodness of fit.

### Hypothesis testing

Significance of the coefficient and t-value from the model testing through a determination of the relationship between variables were presented in Table 5 and Figs 2 and 3. Accordingly, OCB directly affected CTL (β = 0.77, t = 22.31) and CTO (β = 0.42, t = 6.98) confirming H1a and H1b. Findings also indicated that CTL inversely influenced FCC (β = -0.16, t = 2.42). Moreover, CTO directly influenced FCC (β = 0.8, t = 19.72) confirming H2a and H2b.

The OCB’s effect on the FCC confirmed H3a (β = 0.19, t = 2.39). In addition, H3b as an indirect effect and mediating role of CTL and CTO on OCB-FCC relationships was also confirmed. From F^2^ values, these two variables indirectly influenced OCB and FCC at weak and moderate levels, respectively (Table 6).

## Discussion

This Iranian study was conducted in the pediatric nursing society to assess any direct and indirect effects of OCB on FCC with the mediating role of multiple commitment (CTL and CTO) using the SEM-PLS technique. Previous studies investigated and confirmed the effect of commitment on OCB as its predictor [61]. Considering that the main aim of this SEM-based study was to investigate the effect of OCB on FCC, commitment was considered a mediator in the hypothesized model and its effect was investigated on the OCB-FCC relationship.

This study is unique and important, because it investigated the relationships between variables, which had not been studied in Iranian nursing. Also, the relationships between the variables were determined based on the SEM-PLS as an accurate approach in the estimation of relationships between variables. Moreover, multiple commitment including CTL and CTO was investigated at the same time and as a mediator.

The first and second hypotheses were related to the effect of OCB on CTO (CO) and CTL. Some studies are available on the relationship between OCB and OC, and other aspects of commitment such as commitment to the leader. Duarte found that OC and nurses’ professional commitment are correlated with their OCB [10]. Huang et al. conducted a multidimensional analysis of such variables as job satisfaction, organizational commitment, and organizational citizenship behavior [5]. They stated that OC along with other variables changed OCB. Another study on the relationship between OCB and OC in Iranian nursing produced similar results [62].

The literature review did not result in a completely similar study regarding the relationship between OCB and CTL. Wang et al. [63], and Nguni et al. [64], in China and Tanzania, respectively showed that transformational leadership, i.e. the followers’ sense of motivation and value towards the leader, influenced OCB. A study conducted on hospital nurses in an Iranian province showed that leadership had a moderate relationship with OCB [62].

According to the research findings, the third hypothesis was confirmed, and some inverse relationships existed between FCC and CTL. In other words, commitment to the leader was reduced through increasing FCC. However, there are different results in some other studies. For example, Farrell and Oczkowski [43], and Lee et al. [52] found that those employees who had positive exchange relationships with their supervisor were more customer-oriented. A study conducted on employees and customers of service organizations in Taiwan showed that transformational leadership directly and indirectly affected customers with the mediating role of perceived support from supervisors [65]. The reason behind emphasizing the nurse-supervisor relationship was its effect to commitment [35].

The inverse relationship between FCC and CTL can be attributed to various reasons. For instance, the low degree of nurses’ commitment to managers can be attributed to managers and supervisors’ expectations of them to participate in the Iran's National Hospital Accreditation Program inflicted by the lack of procedure and outcome standards [66]. In another qualitative study, participants were dissatisfied with the major burden of the accreditation responsibility on nurses [67]. In general, nurses did not feel committed to their managers, who expected them to have active participations in the inappropriate and challenging accreditation program, and perform better concerning their professional tasks such as FCC.

The fourth hypothesis maintained the positive effect of OC on FCC. Hsu et al. showed that OC affected the customer-centered behavior [47]. Mortazavi and Kargozar, in a study on Iranian nurses showed that OC along with other variables such as occupational justice and satisfaction, affected customer orientation of nurses [68]. Customer orientation is a basis for patient/family-centered care [69], and nurses are committed to the healthcare organization and are specifically sensitive in service delivery and try to meet their customers’ needs beyond their usual job descriptions [47].Therefore, the H3a was confirmed and OCB had a positive effect on FCC. Consistently, Hadjali and Salimi [15] showed that OCB affected the customer-centered behavior among nurses, i.e. customer needs and satisfaction, which was in line with the aims of FCC [70]. In other words, OCB influences the customers’ perceived quality and satisfaction [71]. According to data collected on patients and HCWs by Kolade et al., OCB influenced some dimensions of hospital performance such as efficiency, patient satisfaction, and patient choice [1]. Contrary, two other studies on nurses investigated the effect of the patient-centered care or PCC (defined as PFCC in some texts, which includes FCC) on OCB indicating the effect of perceived patient-centered care on OCB [8, 18].

Finally, OCB directly and indirectly predicted FCC (β = 0.7) with the mediating role of multiple commitment (CTL and CTO) in this study. With regard to the obtained effect size, the extent to which CTO (or affective commitment) influenced OCB and FCC was greater than CTL. In other studies, commitment acted as a mediator. For instance, affective commitment had a mediating role in the relationship between organizational justice and occupational performance [68]. In addition, Chang and Chen found that affective commitment had a mediating role in the relationship between high occupational performance and satisfaction of performance [72].

## Conclusion

This study addressed the relationships between those concepts that were not previously investigated simutaneouly. The SEM-based model showed that OCB directly and indirectly, affected FCC with the mediating role of multiple commitment including CTL and CTO. It is worth noting that in this model, CTL and FCC had an inverse relationship attributed to the Iran’s National Hospital Accreditation, which lowered the employees’ commitment to managers. In general, our findings indicated that the empowerment of OCB and OC in the pediatric nursing community improved FCC as an indicator of high-quality care. Nursing managers can use the findings of the current study to strengthen OCB and employees’ commitment to achieve high-quality healthcare service. Researchers believe that the huge workload of nurses and lack of time to fill out the questionnaires could reduce the accuracy of data collection. Further studies on factors that improve OCB and commitment in a healthcare organization aiming at a better use of the findings of the current study are suggested. While this study was conducted on the pediatric nursing community, its results can be generalized to other nursing areas after conducting similar studies in other culture and contexts.

## Supporting information

S1 File A minimal set of data for the study. (SAV)
